# Mycobacterium avium complex pulmonary disease in rheumatoid arthritis-associated interstitial lung disease under non-biologic immunomodulatory therapy: A case report

**DOI:** 10.1097/MD.0000000000048801

**Published:** 2026-05-22

**Authors:** Ruixia Tang, Run Wang, Yongmei Han

**Affiliations:** a Department of Rheumatology, Sir Run Run Shaw Hospital, Zhejiang University School of Medicine, Hangzhou, Zhejiang, People’s Republic of China; b Department of Pathology, Sir Run Run Shaw Hospital, Zhejiang University School of Medicine, Hangzhou, Zhejiang, People’s Republic of China.

**Keywords:** high-resolution computed tomography (HRCT), interstitial pneumonia, *Mycobacterium avium* complex (MAC), nontuberculous mycobacteria (NTM), rheumatoid arthritis (RA)

## Abstract

**Rationale:**

Rheumatoid arthritis (RA) is a well-recognized risk factor for nontuberculous mycobacterial infections, especially among patients receiving glucocorticoids or biological disease-modifying antirheumatic drugs. However, cases of *Mycobacterium avium* complex (MAC) pulmonary disease in RA patients without such immunosuppressive therapies are rarely reported, which challenges the conventional risk stratification.

**Patient concerns:**

A 78-year-old male with a 3-year history of RA and interstitial lung disease (ILD) presented with progressive dyspnea and chest tightness. He had no fever, joint swelling, or typical infection flares. Before admission, he was treated with Tripterygium Glycosides and Iguratimod (non-biologic, non-glucocorticoid agents).

**Diagnosis:**

The patient had chest tightness and weight loss. Chest high-resolution computed tomography showed asymmetric progression of ILD, along with tree-in-bud signs, centrilobular nodules, and suspicious fibrocavities. Bronchoscopy revealed necrotizing granulomatous inflammation, and quantitative metagenomic sequencing of bronchoalveolar lavage fluid confirmed MAC (no drug-resistant genes detected).

**Interventions:**

The patient was put on a 4-drug anti-MAC regimen (rifampicin, azithromycin, ethambutol, amikacin). However, he was lost to follow-up after being transferred to a tuberculosis specialist hospital. He eventually died of unknown causes, and there were prior reports of his nonadherence to treatment.

**Outcomes:**

For RA patients with ILD who show asymmetric imaging progression or discordant inflammatory markers, it is crucial to actively screen for atypical pathogens like MAC, even in the absence of glucocorticoid or biologic exposure. This case highlights the necessity of expanding nontuberculous mycobacterial infection risk assessment beyond traditional immunosuppressive therapies in RA-ILD patients.

**Lessons:**

For patients with autoimmune disease-associated interstitial pneumonia, particularly those with progressive interstitial lung disease (ILD) despite stable autoimmune serology, proactive screening for atypical pathogens such as nontuberculous mycobacteria is critical. When imaging shows asymmetric lesions, tree-in-bud opacities, centrilobular nodules, or fibrocavitary changes, clinicians should prioritize comprehensive etiological evaluation – including bronchoscopy and histopathology – to avoid misdiagnosing these opportunistic infections.

## 1. Introduction:

Nontuberculous mycobacteria (NTM) are environmental organisms found in water and soil, with over 190 species identified to date. These mycobacteria are categorized into rapidly growing or slowly growing types.^[1]^ The *Mycobacterium avium* complex (MAC) (a slowly growing NTM) is the most prevalent opportunistic pathogen, affecting individuals with immunocompromise, underlying medical conditions, or blunt trauma. It can invade the human body via the respiratory tract, gastrointestinal tract, skin, and other routes, with pulmonary infection being the most common manifestation. Rheumatoid arthritis (RA), a common autoimmune disease, is a well-recognized risk factor for NTM infections,^[2]^ particularly in patients receiving immunosuppressive therapies, such as long-term glucocorticoid use or biologic disease-modifying antirheumatic drugs (bDMARDs).

## 2. Case presentation:

A 78-year-old emaciated male with a 3-year history of RA and interstitial pneumonia was managed with Tripterygium Glycosides tablets and Iguratimod tablets. He presented to our hospital with chest tightness and dyspnea. His medical history was notable for the absence of smoking, prolonged outdoor activities (e.g., fishing, gardening), and occupational exposure to metals, chemicals, or other agents linked to pulmonary disorders. He denied fever, joint swelling, skin rashes, digital ulcers, or Raynaud phenomenon. On admission, his weight was 49.1 kg, body mass index (BMI) was 18.8 kg/m^2^, and vital signs were within normal limits. Lung auscultation revealed fine crackles at the right base.

Laboratory tests revealed erythrocyte sedimentation rate of 91 mm/h, hypersensitive C-reactive protein of 41.7 mg/L, albumin of 26.6 g/L, rheumatoid factor of 225.9 IU/mL, alkaline phosphatase of 277U/L, gamma-glutamyl transpeptidase of 61U/L, positive anti-cyclic citrullinated peptide antibodies, positive tuberculosis infection T-cells, negative G tests and *Aspergillus* galactomannan tests, and negative human immunodeficiency virus. Serum creatinine, lymphocyte count, CD4^+^ T-cell/CD8^+^ T-cell were within the normal range.

**Figure 1.**
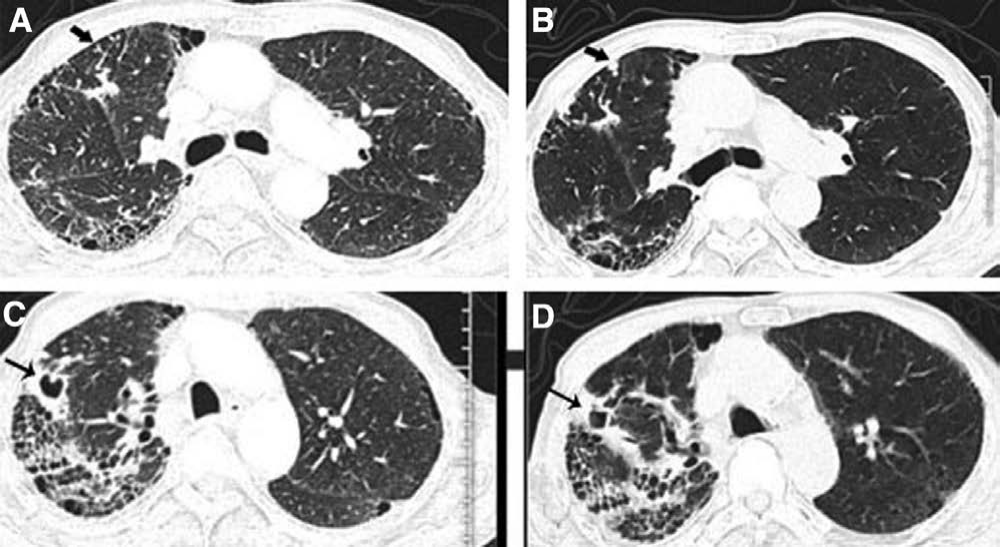
Chest HRCT reveals signs of pulmonary fibrosis, such as interstitial infiltrates with reticular patterns and areas of ground-glass opacities in the right lung. In the upper lung fields, it shows (A) a tree-in-bud pattern in the right lung (thick black arrows) and (B) centrilobular nodules at the original tree-in-bud site (thick black arrows). In the middle lung fields, (C), (D) thick-walled fibrous cavity lesions are visible (thin black arrows). HRCT = high-resolution computed tomography..

Joint ultrasonography revealed synovial thickening and active inflammation in multiple joints, with bone erosion. High-resolution computed tomography (HRCT) of the chest indicated progression of usual interstitial pneumonia. Pulmonary function tests indicate moderate diffusion impairment (forced vital capacity 1.92 L, diffusing capacity of the lung for carbon monoxide 3.33 mmol/min/Kpa, diffusing capacity of the lung for carbon monoxide as a percentage of the expected value 44.5%). Bronchoscopy and lung biopsy showed necrotizing granulomatous inflammation in the right upper lobe (Fig. 1), no malignant cells in the bronchial lavage fluid or brushings. The acid-fast staining, the detection of *Mycobacterium tuberculosis* DNA, the bacterial and fungal culture results of the bronchoalveolar lavage fluid were negative. MAC (Sequence number 62, relative abundance 5.16%, Q index 43-more than 92.10% of the similar specimens) was detected using the quantitative metagenomic sequencing (Q-mNGS) quantitative metagenomic sequencing method, and no drug-resistant genes were detected. Which led to the diagnosis of MAC pulmonary disease (PD).

**Figure 2.**
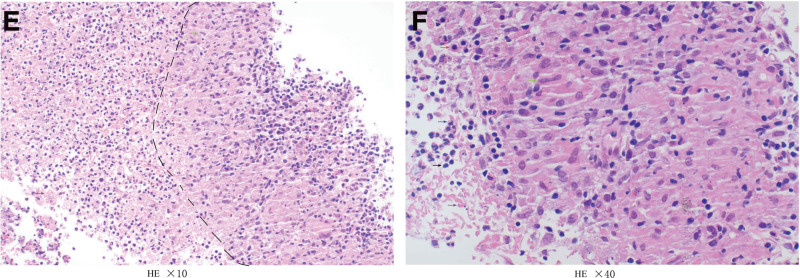
At low power (×10), the dotted line demarcates granulomatous inflammation on the right, the left side shows inflammatory cells with extensive necrotic tissue (E). At high power (×40), the granulomatous inflammation site reveals abundant epithelioid cells and multinucleated giant cells (F).

On May 6, 2023, the patient was treated with a 4-drug regimen of rifampicin, azithromycin, ethambutol, and amikacin. Specifically: Rifampicin at 0.6 g orally once daily, Azithromycin at 500 mg orally once daily, Ethambutol at 0.75 g orally once daily, and Amikacin at 0.4 g intravenous drip, 3 times a week for 12 weeks (the entire medication course continued until sputum culture turned negative for at least 1 year). During the medication period, it is recommended that patients have their liver and kidney functions checked and ocular and auditory toxicity evaluated every 2 weeks. Along with continued immunosuppressive therapy with Tripterygium Glycosides tablets at 10mg orally 3 times a day and Iguratimod Tablets at 25 mg orally twice a day to control RA activity, supplemented with gastroprotective and hepatoprotective treatments. During the follow-up period, we found that the patient’s alkaline phosphatase and gamma-glutamyl transpeptidase levels remained relatively stable. A follow-up chest computed tomography showed progression in the right upper lung (Fig. 2B). He did not have any vision or hearing problems, and the joint swelling and pain were not significant. After taking the medication, he experienced stomach discomfort and poor appetite. We added treatments for protecting the stomach and promoting digestion, continued with the liver-protecting, anti-NTM infection and anti-RA treatment. On September 20, 2023, the patient visited a tuberculosis specialist hospital and did not come to our hospital for follow-up. During the telephone follow-up, the patient informed that due to gastrointestinal discomfort, he had not received regular anti-NTM treatment and discontinued in November 2023. On April 8, 2024, the reexamination of chest computed tomography at that hospital indicated that the lung lesion had progressed. Unfortunately, the patient died, and the attribution of death could not be carried out due to lack of patient follow-up data.

Reviewing the patient’s imaging findings, a chest HRCT report suggested usual interstitial pneumonia (Fig. 2). Tree-in-bud patterns (Fig. 2A), pulmonary mass lesion (Fig. 2C, Fig. 2D), bronchiectasis (BR), and suspicious fibrous cavity disease (Fig. 2A, Fig. 2B) were found in the right lung lobe. As the disease progressed, the tree-in-bud pattern in the right lung evolved into centrilobular nodules (Fig. 2B).

## 3. Discussion

This report details a case of MAC-PD in a patient with RA-associated interstitial lung disease (RA-ILD). The patient presented with chest tightness and dyspnea, with no signs of infection. The patient had previously received treatment with Tripterygium Glycosides tablets and Iguratimod tablets, without exposure to glucocorticoid or biologic therapy. Given the observed disparity between elevated inflammatory markers and mild joint symptoms, the asymmetric progression of bilateral interstitial pneumonia, the presence of a pulmonary mass lesion, and the suspicious fibrous cavity seen on HRCT imaging, we suspected additional lung pathologies beyond RA-ILD.

According to China’s 2020 Guidelines for the diagnosis and treatment of NTM, the patient experienced chest tightness and weight loss. Chest HRCT showed asymmetric progressive interstitial lung disease (ILD) with tree-in-bud signs, centrilobular nodules, and suspicious fibrocavities. A lung biopsy revealed necrotizing granulomatous inflammation, and Q-mNGS of bronchoalveolar lavage fluid (BALF) confirmed the presence of MAC, meeting the diagnostic criteria for NTM-PD (Table 1). Clinically, since the patient did not expectorate, repeated bronchoscopies for BALF collection were not feasible in the short term, leading to the failure to obtain multiple samples. To ensure sample quality and result credibility, strict disinfection and aseptic procedures were followed during collection and submission to prevent soil and water contamination. Detection accuracy was enhanced by filtering background microbial libraries, removing low-abundance sequences, and retaining highly reliable reads. Considering the Q-mNGS results, clinical symptoms, imaging findings, results of tuberculosis infection T-cells, and pathology, and ruling out bacterial, fungal, tuberculosis, and tumor diseases, MAC was identified as the pathogenic organism. A comprehensive assessment by respiratory experts confirmed the diagnosis of MAC-PD. Regrettably, NTM infection was not considered during the initial diagnostic workup, so BALF was not cultured using NTM-specific media. This resulted in negative culture results and the inability to perform drug susceptibility testing, ultimately relying on Q-mNGS findings for diagnosis. A study evaluating the diagnostic efficacy of high-throughput sequencing (HTS) versus mycobacterial culture for NTM-PD demonstrated that HTS exhibits higher sensitivity and comparable specificity to mycobacterial culture for NTM-PD diagnosis.^[3]^ Additionally, the 2025 Chinese Expert Consensus on NTM-PD recommends the use of molecular detection methods, such as HTS, to aid in the diagnosis of NTM-PD.^[2]^

**Table 1:** Diagnostic criteria for NTM-PD.

Clinical	Pulmonary and/or Systemic Symptoms
Imaging features	Cavities, multifocal bronchiectasis and multiple small nodular lesions
	And exclude other lung diseases
Microbiology	1. Two separate sputum specimens tested positive for NTM culture, identifying the same pathogen; and/or NTM molecular biology tests also confirmed it.
	2. One positive result for NTM culture and/or molecular biology testing in bronchial lavage fluid or bronchoalveolar lavage fluid.
	3. Bronchoscopic or other invasive lung biopsy showed characteristic NTM histopathological changes (granulomatous inflammation or positive acid - fast staining), and NTM culture and/or molecular biological testing was positive.
	4. Lung biopsy via bronchoscopy or other methods showed characteristic NTM histopathological changes (granulomatous inflammation or positive acid - fast staining), and NTM culture and/or molecular biological testing was positive in one or more sputum specimens, bronchial lavage fluid, or bronchoalveolar lavage fluid.

The diagnosis of NTM-PD requires simultaneous fulfillment of clinical symptoms, imaging features, and at least 1 microbiologic criterion (specimens must exclude exogenous contamination), with exclusion of other respiratory diseases.^[2]^

NTM = nontuberculous mycobacteria, PD = pulmonary disease.

Increasing clinical attention has focused on NTM infections in patients with RA. Studies indicate a significantly higher prevalence of NTM infections in RA cohorts compared to the general population, most attribute this risk to long-term use of glucocorticoids or bDMARDs: particularly tumor necrosis factor-α (TNF-α) inhibitors such as infliximab,^[4]^ adalimumab,^[5]^ and etanercept. A 2007 review of FDA data on NTM infections found that approximately 73.70% of NTM-infected patients had RA, with most cases linked to infliximab, etanercept, and adalimumab. Notably, many of these patients were also receiving concurrent prednisone or methotrexate.^[6]^ The underlying mechanism is thought to involve TNF-α inhibitors suppressing the production and signaling of inflammatory cytokines,^[5]^ as well as downregulating adhesion molecules (e.g., intercellular adhesion molecule 1),^[7]^ thereby increasing susceptibility to NTM infections. A study investigating the correlation between NTM infections and drug therapy indicates that baseline glucocorticoid use is a risk factor for NTM-PD, whereas conventional disease-modifying antirheumatic drugs (DMARDs) do not increase this risk.^[8]^ Few cases of NTM infections following conventional DMARDs use have been reported (see Table 2). Notably, the patient in our report had no history of bDMARD or glucocorticoid treatment, distinguishing this case from previously reported instances.

**Table 2:** Summary of cases of NTM-PD in a patient with RA.

	Age (yr)/ Sex	Disease Duration (yr)	symptom	Basic diseases	Immunosuppressive drugs	CT manifestation
H. Okubo et al^[3]^	67/F	47	Polyarthralgia	NA	Infliximab (DC)	A small nodular shadow
H. Nakahara et al^[9]^	63/F	19	Polyarthralgia cough and sputum	MPP	Tocilizumab (DC)	consolidation, cavity formation, centrilobular nodules, and bronchiectasis
Ho Namkoong et al^[10]^	63/F	NA	Polyarthralgia hemoptysis	NA	Tocilizumab (DC/RD)	a single cavitary lesion
Hiromu Tanaka et al^[11]^	62/F	NA	hemosputum	T2DM	Abatacept (Diagnosing RA after MAC infection)	cavitary lesions
Kenichiro Yaita MD et al^^[12]^^	78/F	11	Fever osteomyelitis	NA	Prednisolone; bucillamine	small bandlike shadows on the right lower lobe

DC = drug discontinuance, F = female, MAC = *Mycobacterium avium* complex, MPP = mycoplasma pneumonia, NA = not available, PD = pulmonary disease, RA = rheumatoid arthritis, RD = reusing drugs, T2DM = type 2 diabetes mellitus, yr = year.

NTM infections can involve the lungs, skin, and joints, with respiratory manifestations being the most common.^[2]^ NTM-PD occurs more frequently in immunocompromised hosts and individuals with underlying lung diseases. Notably, immunosuppressed patients face an elevated risk of disseminated infections.^[13]^ A retrospective study in Japan identified RA as a systemic risk factor for pulmonary NTM infection. A low BMI is both a characteristic feature of NTM-PD patients and a marker of poor prognosis. Specifically in RA patients, MAC infection has been strongly associated with low peripheral blood lymphocyte counts (< 1500/μL) and low body weight (BMI < 18.0 kg/m^2^).^[14]^ In this case, the patient presented with several recognized risk factors for NTM infection: advanced age, a lean physique, RA, interstitial pneumonia, and the use of immunosuppressive medications.

Patients with RA face an elevated risk of NTM-PD, though the precise underlying causes and mechanisms remain incompletely understood. RA, a common immune-mediated disease, frequently involves the lungs-including interstitial pneumonia, BR, and chronic obstructive PD.^[15]^ Structural lung abnormalities in RA may predispose individuals to NTM colonization and infection. A cohort study examining RA-associated lung disease (RA-LD) and severe infection risk found that RA-LD, particularly RA-ILD, correlates with an increased risk of serious systemic infections (including NTM), while RA-associated BR is linked to higher rates of pulmonary infections (including NTM).^[16]^ Additionally, disease-related immune dysfunction may contribute to NTM susceptibility, especially via T-cell repertoire restriction.^[17]^ For example, RA patients often exhibit expanded populations of CD28-null T-cells, which downregulate the function of antigen-presenting dendritic cells,^[18]^ impairing timely T-cell activation and thus increasing infection risk. Finally, immunosuppressive therapies (such as glucocorticoids and bDMARDs) —further amplify this risk.^[19]^

The radiological manifestations of NTM-PD primarily fall into 2 patterns: fibrocavitary and nodular bronchiectatic, they can overlap.^[20]^ While lung involvement can affect any lobe or segment, the upper lobes, right middle lobe, and lingula are most frequently involved. Common radiological findings include tree-in-bud opacities, centrilobular nodules, BR (including capillary BR), fibrocavitary lesions, patchy consolidations, ground-glass opacities, and emphysema. These features are diverse and nonspecific, systemic symptoms are often subtle (Table 2). This combination makes diagnosis challenging, frequently leading to misdiagnosis or delayed treatment. This is particularly problematic in RA patients with interstitial pneumonia, where NTM-related imaging changes may be misattributed to progression of ILD, resulting in inappropriate management.

Histopathologically, NTM-PD is characterized by granulomatous inflammation of the airway walls, which is visible on imaging as tree-in-bud and nodular changes. Chronic inflammatory granulomas can obstruct airways, driving BR. The co-occurrence of centrilobular nodules and BR is a hallmark pathological feature of NTM-PD. Inflammatory mediators like TNF-α can also promote tissue necrosis and cavitation, manifesting radiologically as fibrocavitary lesions.^[21]^ The false-negative tissue acid-fast staining in this case may stem from 3 key factors: strain characteristics, pathogen burden and tissue processing. Unlike *M tuberculosis*, some MAC strains exhibit weak acid-fastness, increasing false-negative risk. Low bacterial load in tissue samples can reduce detection sensitivity. Concentrating the specimens before hematoxylin-eosin staining has been shown to enhance the positive rate of acid-fast staining.^[22]^ Delayed staining or prolonged storage degrades acid-fast components in NTM cell walls, impairing stain binding.

Consensus on treating RA complicated by NTM infection remains elusive. Some studies suggest antirheumatic therapy does not exacerbate NTM progression. Mori et al documented favorable outcomes with biologic agents in patients with concurrent NTM infection. A single-center retrospective cohort study in Japan found no association between bDMARDs and worse NTM-PD prognosis.^[8]^ However, other research highlights potential risks of biologics (particularly TNF-α inhibitors) in NTM infection and outcomes.^[23,24]^ Japanese guidelines explicitly recommend avoiding TNF-α inhibitors in patients with NTM infection.^[25]^ Notably, Greenberg et al reported no synergistic infection risk with combined methotrexate and TNF antagonists.^[26]^ In the context of anti-NTM therapy, the 2025 Chinese Expert Consensus underscores the clinical importance of managing the comorbidity of NTM-PD and BR. For patients with NTM-PD complicated by BR, anti-NTM treatment is advised when cavitary lesions are detected on imaging. Moreover, the consensus stresses that for patients with moderate to severe BR without confirmed pathogen culture results, routine coverage against *Pseudomonas* is recommended. In such cases, patients with MAC-PD should undergo anti-NTM treatment due to the presence of adverse prognostic factors, including advanced age, low body weight, BR, and fibrotic cavities. Macrolides are the cornerstone of NTM-PD therapy, particularly for MAC-induced PD.^[2]^ Both the American Thoracic Society and British Thoracic Society guidelines recommend a macrolide-based 3-drug regimen: a macrolide, ethambutol, and a rifamycin.^[27,28]^ In this context, the inclusion of amikacin in the treatment regimen is justified by 2 key considerations: Anti-NTM efficacy: amikacin is a first-line injectable agent for NTM-PD, (particularly in patients with cavitary lesions) a poor prognostic factor; *Pseudomonas* coverage: amikacin demonstrates broad-spectrum activity against *Pseudomonas aeruginosa*, aligning with the recommendation for empiric coverage in severe BR. Based on domestic and international guidelines and China’s clinical practice, the recommended treatment regimen for MAC-PD with fibrotic cavities or BR is as follows: Macrolide: Azithromycin 500–600 mg/day, or clarithromycin 500–1000 mg/day (500 mg/day if body weight < 50 kg).

Rifampicin: 450–600 mg/day (450 mg/day if body weight < 50 kg). Ethambutol: 15 mg/kg/day(oral). Amikacin:20–25 mg/kg/day (adults, maximum 1.0 g/day), administered via intramuscular injection, intravenous infusion, or nebulization. Duration: amikacin is typically used for the first 3 months, and the entire regimen is continued until sputum cultures remain negative for at least 1 year.

For the treatment of RA, the patient’s polyarticular swelling and pain were mild, and elevated inflammatory markers were likely attributable to MAC-PD. Despite a disease activity score 28 score of 4.35, treatment with Tripterygium Glycosides tablets and Iguratimod tablets was continued without dose adjustments. Tripterygium Glycosides, a commonly used tradition Chinese herbal medicine, is widely applied in autoimmune diseases due to its potent anti-inflammatory and immunomodulatory properties.^[29]^ Similarly, iguratimod (a conventional synthetic DMARD) is also a standard therapy for RA.^[30]^ To date, no cases of NTM infection have been reported with the co-administration of these 2 agents. Regarding drug interactions, rifampicin is a potent inducer of the cytochrome P450 enzyme system, primarily accelerating the metabolism of drugs metabolized by enzymes such as cytochrome P3A4.^[31]^ Concomitant use of rifampicin with Tripterygium glycosides increases the latter’s metabolic rate, leading to reduced serum concentrations. However, given the patient’s impaired liver function, no dose adjustment of Tripterygium glycosides was made.

The patient sought care at a tuberculosis-specialized hospital and did not return to our institution for follow-up. Attempts to obtain follow-up data via telephone were unsuccessful due to the patient’s noncooperation, and detailed follow-up information could not be obtained. The patient eventually passed away from an unknown cause. Given the incomplete follow-up, this case cannot provide insights into drug selection, therapeutic efficacy, adverse drug reactions, serial imaging changes of MAC-PD, or disease prognosis.

Unlike tuberculosis,^[26]^ NTM infections are frequently acquired during immunosuppressive therapy,^[5]^ and optimal preventive measures for such infections in patients remain unclear. Research has linked RA-LD and RA-ILD to an elevated risk of severe infections; whether treating RA-ILD reduces NTM infection risk requires further investigation.^[16]^ A South Korean clinical study found hydroxychloroquine exerts a protective effect against NTM infection-related mortality, suggesting this well-tolerated, effective RA medication may have potential for preventing NTM infections.^[32]^ However, larger sample sizes and additional studies are needed to confirm this finding. Currently, the primary focus is maintaining vigilance for NTM infections in patients with rheumatic diseases.

## 4. Conclusion:

This case describes an elderly patient with RA-ILD who developed secondary pulmonary MAC infection without exposure to glucocorticoids or biologic agents. While the exact mechanisms underlying secondary NTM infections in RA remain unclear, accumulating case reports and studies suggest a strong link to immunosuppression from glucocorticoids and biologic therapies. This case highlights that RA-related immune dysfunction, structural lung disease, emaciation, and advanced age may act as independent risk factors for NTM infection-challenging the traditional view that “only RA patients on glucocorticoid or biologic therapy have an increased risk of MAC infection.”

For patients with autoimmune diseases complicated by interstitial pneumonia, especially those with progressive ILD despite stable autoimmune activity, active screening for atypical pathogens like NTM is critical. When imaging shows asymmetric pulmonary lesions, tree-in-bud signs, centrilobular nodules, or fibrocavitary changes, clinicians should prioritize comprehensive etiological investigations-including bronchoscopy and histopathology-to avoid misdiagnosing opportunistic infections.

Notably, repeated sampling for culture and testing was not performed. Prior to sample submission, NTM infection was not suspected, and specialized culture media or conditions were not specified-leading to negative culture results, failure to complete drug susceptibility testing, and a lack of guidance for subsequent treatment. Additionally, the case was lost to full follow-up, limiting definitive insights into therapeutic efficacy, longitudinal biomarker trends, and disease prognosis. Existing data are also limited to lymphocyte counts and TBNK (T cells, B cells, and natural killer cells ) subset analyses, with insufficient immunophenotyping evidence to fully characterize the patient’s immune status; this may compromise the generalizability of the conclusions.

For RA patients with MAC-PD, management must balance anti-infective therapy and control of the underlying disease: MAC-PD requires a macrolide-based combination regimen (e.g., azithromycin, ethambutol, rifampicin), with concurrent management of associated BR per clinical guidelines. RA can be controlled using non-biologic DMARDs. Close monitoring of drug interactions and infection progression is also critical. The mechanisms underlying RA-related susceptibility to NTM, as well as the role of autoimmune dysfunction in NTM infection, require further investigation.

## Author contributions

**Data curation:** Ruixia Tang.

**Formal analysis:** Ruixia Tang, Run Wang, Yongmei Han.

**Writing—original draft:** Ruixia Tang.

**Writing—review & editing:** Yongmei Han.
